# 2,4-Bis(4-but­oxy­phen­yl)-3-aza­bicyclo­[3.3.1]nonan-9-one

**DOI:** 10.1107/S1600536811005058

**Published:** 2011-02-16

**Authors:** P. Parthiban, V. Ramkumar, Yeon Tae Jeong

**Affiliations:** a Department of Image Science and Engineering, Pukyong National University, Busan 608 737, Republic of Korea; b Department of Chemistry, IIT Madras, Chennai, TamilNadu, India

## Abstract

In the title compound, C_28_H_37_NO_3_, a crystallographic mirror plane bis­ects the mol­ecule (one half-mol­ecule in the asymmetric unit). The title compound exists in a twin-chair conformation with an equatorial orientation of the 4-but­oxy­phenyl groups. Both sides of the secondary amino group carry the 4-but­oxy­phenyl groups at an angle of 38.54 (3)° with respect to one another.

## Related literature

For the synthesis and biological activity of 3-aza­bicyclo­[3.3.1] nonan-9-ones, see: Jeyaraman & Avila (1981 ▶); Barker *et al.* (2005 ▶); Parthiban *et al.* (2009*a* ▶, 2010*b* ▶,*c* ▶); Cox *et al.* (1985 ▶). For related structures, see: Parthiban *et al.* (2009*b* ▶,*c* ▶, 2010*a* ▶); Smith-Verdier *et al.* (1983 ▶); Padegimas & Kovacic (1972 ▶). For ring puckering parameters, see: Cremer & Pople (1975 ▶); Nardelli (1983 ▶).
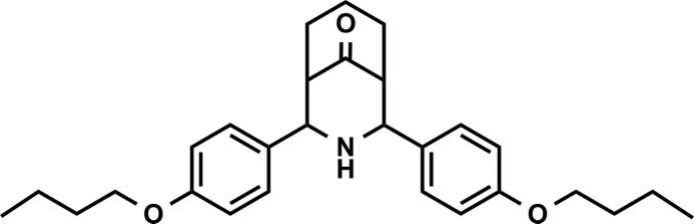


## Experimental

### 

#### Crystal data

C_28_H_37_NO_3_
*M* *_r_* = 435.59
Orthorhombic, 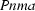

*a* = 7.7780 (5) Å
*b* = 31.457 (2) Å
*c* = 9.9560 (6) Å
*V* = 2436.0 (3) Å^3^
*Z* = 4
Mo *K*α radiation
μ = 0.08 mm^−1^
*T* = 298 K
0.35 × 0.28 × 0.25 mm

#### Data collection

Bruker APEXII CCD area-detector diffractometer
Absorption correction: multi-scan (*SADABS*; Bruker, 2004 ▶) *T*_min_ = 0.974, *T*_max_ = 0.981
10360 measured reflections
2991 independent reflections
1900 reflections with *I* > 2σ(*I*)
*R*_int_ = 0.025

#### Refinement

*R*[*F* ^2^ > 2σ(*F* ^2^)] = 0.056
*wR*(*F* ^2^) = 0.163
*S* = 1.02
2991 reflections
155 parameters
H atoms treated by a mixture of independent and constrained refinement
Δρ_max_ = 0.32 e Å^−3^
Δρ_min_ = −0.18 e Å^−3^

### 

Data collection: *APEX2* (Bruker, 2004 ▶); cell refinement: *APEX2* and *SAINT-Plus* (Bruker, 2004 ▶); data reduction: *SAINT-Plus* and *XPREP* (Bruker, 2004 ▶); program(s) used to solve structure: *SHELXS97* (Sheldrick, 2008 ▶); program(s) used to refine structure: *SHELXL97* (Sheldrick, 2008 ▶); molecular graphics: *ORTEP-3* (Farrugia, 1997 ▶); software used to prepare material for publication: *SHELXL97*.

## Supplementary Material

Crystal structure: contains datablocks global, I. DOI: 10.1107/S1600536811005058/bq2279sup1.cif

Structure factors: contains datablocks I. DOI: 10.1107/S1600536811005058/bq2279Isup2.hkl

Additional supplementary materials:  crystallographic information; 3D view; checkCIF report

## Figures and Tables

**Fig. 1.**
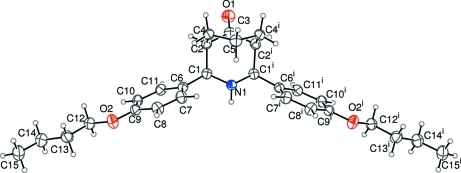
Anisotropic displacement representation of the molecule with 30% probability ellipsoids. Symmetry code: (i) x, -y+1/2, z.

## supplementary crystallographic information

### Comment

Naturally abundant diterpenoid/norditerpenoid alkaloids contain the 3-azabicyclononane nucleus, which is an important class of pharmacophore due to its broad spectrum of biological activities such as antibacterial, antimycobacterial, antifungal, anticancer, antitussive, anti-inflammatory, sedative, antipyretic and calcium antagonistic activity (Jeyaraman & Avila, 1981; Barker *et al.*, 2005; Parthiban *et al.*, 2009*a*, 2010*b*, 2010*c*). Its biological significant prompted the medicinal chemists to synthesize some structural analogs. Since the stereochemistry plays an important role in biological actions, it is important to establish the stereochemistry of the synthesized bio-potent molecules. For the synthesized title compound, several stereomers are possible with conformations such as chair-chair (Parthiban *et al.*, 2009*b*, 2009*c*, 2010*a*; Cox *et al.*, 1985), chair-boat (Smith-Verdier *et al.*, 1983) and boat-boat (Padegimas & Kovacic, 1972). Hence, the title crystal was undertaken for this study to explore its stereochemistry, unambiguously.

The analysis of torsion angles, asymmetry parameters and puckering parameters calculated for the title compound shows that the piperidine ring adopts a near ideal chair conformation. According to Cremer & Pople, the total puckering amplitude, Q_T_ is -0.613 (2) Å and the phase angle θ is 178.67 (19)° (Cremer & Pople, 1975). The smallest displacement asymmetry parameters q_2_ and q_3_ are 0.005 (2) and -0.612 (2)°, respectively (Nardelli, 1983). However, the cyclohexane ring deviates from the ideal chair conformation according to Cremer and Pople by Q_T_ = 0.573 (2) and θ = 16.1 (2)° (Cremer & Pople, 1975) as well as Nardelli by q_2_ = 0.158 (2) and q_3_ = 0.550 (2)° (Nardelli, 1983). Hence, the title compound C_28_H_37_NO_3_, exists in a twin-chair conformation with equatorial orientation of the 4-butoxyphenyl groups on both sides of the secondary amino group on the heterocycle. The aryl groups are orientated at an angle of 38.54 (3)° to each other. The torsion angle of C3—C2—C1—C6 and its mirror image is 176.03 (4)°. The crystal packing is stabilized by weak van der Waals interactions.

### Experimental

The title compound was synthesized by a modified and an optimized Mannich condensation in one-pot, using 4-butoxybenzaldehyde (0.1 mol, 17.82 g/17.29 ml), cyclohexanone (0.05 mol, 4.90 g/5.18 ml) and ammonium acetate (0.075 mol, 5.78 g) in a 50 ml of absolute ethanol. The mixture was gently warmed on a hot plate at 303–308 K (30–35° C) with moderate stirring till the complete consumption of the starting materials, which was monitored by TLC. At the end, the crude azabicyclic ketone was separated by filtration and gently washed with 1:5 cold ethanol-ether mixture. X-ray diffraction quality crystals of the title compound were obtained by slow evaporation from ethanol.

### Refinement

The nitrogen H atom was located in a difference Fourier map and refined isotropically. Other hydrogen atoms were fixed geometrically and allowed to ride on the parent carbon atoms with aromatic C—H = 0.93 Å, aliphatic C—H = 0.98Å and methylene C—H = 0.97 Å. The displacement parameters were set for phenyl, methylene and aliphatic H atoms at *U*_iso_(H) = 1.2*U*_eq_(C).

### Crystal data

**Table:**

C_28_H_37_NO_3_	*F*(000) = 944
*M**_r_* = 435.59	*D*_x_ = 1.188 Mg m^−^^3^
Orthorhombic, *P**n**m**a*	Mo *K*α radiation, λ = 0.71073 Å
Hall symbol: -P 2ac 2n	Cell parameters from 4431 reflections
*a* = 7.7780 (5) Å	θ = 3.3–26.9°
*b* = 31.457 (2) Å	µ = 0.08 mm^−^^1^
*c* = 9.9560 (6) Å	*T* = 298 K
*V* = 2436.0 (3) Å^3^	Block, colorless
*Z* = 4	0.35 × 0.28 × 0.25 mm

### Data collection

**Table:**

Bruker APEXII CCD area-detector diffractometer	2991 independent reflections
Radiation source: fine-focus sealed tube	1900 reflections with *I* > 2σ(*I*)
graphite	*R*_int_ = 0.025
phi and ω scans	θ_max_ = 28.3°, θ_min_ = 2.2°
Absorption correction: multi-scan (*SADABS*; Bruker, 2004)	*h* = −10→9
*T*_min_ = 0.974, *T*_max_ = 0.981	*k* = −21→41
10360 measured reflections	*l* = −11→13

### Refinement

**Table:**

Refinement on *F*^2^	Primary atom site location: structure-invariant direct methods
Least-squares matrix: full	Secondary atom site location: difference Fourier map
*R*[*F*^2^ > 2σ(*F*^2^)] = 0.056	Hydrogen site location: inferred from neighbouring sites
*wR*(*F*^2^) = 0.163	H atoms treated by a mixture of independent and constrained refinement
*S* = 1.02	*w* = 1/[σ^2^(*F*_o_^2^) + (0.0606*P*)^2^ + 1.2024*P*] where *P* = (*F*_o_^2^ + 2*F*_c_^2^)/3
2991 reflections	(Δ/σ)_max_ < 0.001
155 parameters	Δρ_max_ = 0.32 e Å^−^^3^
0 restraints	Δρ_min_ = −0.18 e Å^−^^3^

### Special details

**Table:**

Geometry. All e.s.d.'s (except the e.s.d. in the dihedral angle between two l.s. planes) are estimated using the full covariance matrix. The cell e.s.d.'s are taken into account individually in the estimation of e.s.d.'s in distances, angles and torsion angles; correlations between e.s.d.'s in cell parameters are only used when they are defined by crystal symmetry. An approximate (isotropic) treatment of cell e.s.d.'s is used for estimating e.s.d.'s involving l.s. planes.
Refinement. Refinement of *F*^2^ against ALL reflections. The weighted *R*-factor *wR* and goodness of fit *S* are based on *F*^2^, conventional *R*-factors *R* are based on *F*, with *F* set to zero for negative *F*^2^. The threshold expression of *F*^2^ > σ(*F*^2^) is used only for calculating *R*-factors(gt) *etc*. and is not relevant to the choice of reflections for refinement. *R*-factors based on *F*^2^ are statistically about twice as large as those based on *F*, and *R*- factors based on ALL data will be even larger.

### Fractional atomic coordinates and isotropic or equivalent isotropic displacement parameters (Å^2^)

**Table:**

	*x*	*y*	*z*	*U*_iso_*/*U*_eq_	
C1	1.1848 (3)	0.71161 (6)	0.10972 (19)	0.0455 (5)	
H1	1.2239	0.7136	0.2031	0.055*	
C2	1.3471 (3)	0.71050 (6)	0.0188 (2)	0.0487 (5)	
H2	1.4165	0.6857	0.0432	0.058*	
C3	1.4496 (4)	0.7500	0.0466 (3)	0.0494 (7)	
C4	1.3130 (3)	0.70937 (6)	−0.1338 (2)	0.0523 (5)	
H4A	1.4208	0.7044	−0.1801	0.063*	
H4B	1.2372	0.6857	−0.1536	0.063*	
C5	1.2325 (4)	0.7500	−0.1884 (3)	0.0547 (7)	
H5A	1.1109	0.7500	−0.1667	0.066*	
H5B	1.2429	0.7500	−0.2855	0.066*	
C6	1.0781 (3)	0.67181 (6)	0.09708 (18)	0.0440 (4)	
C7	0.9491 (3)	0.66660 (6)	0.0020 (2)	0.0542 (5)	
H7	0.9213	0.6891	−0.0544	0.065*	
C8	0.8618 (3)	0.62882 (7)	−0.0105 (2)	0.0560 (5)	
H8	0.7765	0.6260	−0.0753	0.067*	
C9	0.9001 (3)	0.59496 (6)	0.0730 (2)	0.0495 (5)	
C10	1.0237 (3)	0.59983 (6)	0.1706 (2)	0.0530 (5)	
H10	1.0487	0.5775	0.2288	0.064*	
C11	1.1108 (3)	0.63806 (6)	0.1821 (2)	0.0500 (5)	
H11	1.1936	0.6411	0.2489	0.060*	
C12	0.8378 (3)	0.52285 (6)	0.1337 (2)	0.0601 (6)	
H12A	0.8154	0.5296	0.2271	0.072*	
H12B	0.9567	0.5139	0.1253	0.072*	
C13	0.7190 (3)	0.48792 (7)	0.0868 (3)	0.0663 (6)	
H13A	0.6013	0.4978	0.0945	0.080*	
H13B	0.7413	0.4825	−0.0075	0.080*	
C14	0.7355 (4)	0.44743 (7)	0.1615 (3)	0.0752 (7)	
H14A	0.7082	0.4523	0.2553	0.090*	
H14B	0.8538	0.4377	0.1566	0.090*	
C15	0.6187 (4)	0.41318 (8)	0.1070 (3)	0.0882 (9)	
H15A	0.5020	0.4231	0.1080	0.132*	
H15B	0.6283	0.3882	0.1618	0.132*	
H15C	0.6517	0.4065	0.0165	0.132*	
N1	1.0856 (3)	0.7500	0.0803 (2)	0.0456 (5)	
O1	1.5979 (3)	0.7500	0.0826 (2)	0.0692 (6)	
O2	0.8061 (2)	0.55880 (5)	0.05158 (16)	0.0662 (5)	
H1N	0.991 (4)	0.7500	0.127 (3)	0.052 (9)*	

### Atomic displacement parameters (Å^2^)

**Table:**

	*U*^11^	*U*^22^	*U*^33^	*U*^12^	*U*^13^	*U*^23^
C1	0.0549 (11)	0.0463 (10)	0.0354 (10)	0.0017 (9)	−0.0014 (8)	0.0015 (8)
C2	0.0523 (11)	0.0457 (10)	0.0480 (11)	0.0066 (9)	−0.0010 (9)	0.0022 (8)
C3	0.0480 (16)	0.0612 (17)	0.0392 (15)	0.000	−0.0013 (13)	0.000
C4	0.0609 (12)	0.0500 (11)	0.0459 (11)	−0.0011 (9)	0.0079 (10)	−0.0064 (9)
C5	0.0652 (19)	0.0616 (18)	0.0374 (15)	0.000	−0.0012 (14)	0.000
C6	0.0516 (10)	0.0444 (10)	0.0360 (10)	0.0038 (8)	0.0046 (8)	0.0018 (8)
C7	0.0670 (13)	0.0523 (12)	0.0434 (11)	0.0004 (10)	−0.0066 (10)	0.0120 (9)
C8	0.0617 (12)	0.0598 (13)	0.0465 (12)	−0.0056 (10)	−0.0106 (10)	0.0068 (9)
C9	0.0540 (11)	0.0458 (10)	0.0487 (12)	0.0017 (9)	0.0047 (9)	0.0021 (9)
C10	0.0566 (12)	0.0458 (11)	0.0565 (13)	0.0079 (9)	−0.0016 (10)	0.0125 (9)
C11	0.0534 (11)	0.0511 (11)	0.0456 (11)	0.0063 (9)	−0.0059 (9)	0.0055 (9)
C12	0.0604 (13)	0.0494 (12)	0.0704 (15)	0.0043 (10)	0.0027 (12)	0.0078 (10)
C13	0.0629 (13)	0.0600 (14)	0.0761 (17)	−0.0033 (11)	0.0005 (12)	0.0103 (12)
C14	0.0776 (16)	0.0589 (14)	0.0893 (19)	0.0063 (12)	0.0013 (15)	0.0050 (13)
C15	0.104 (2)	0.0565 (14)	0.104 (2)	−0.0075 (14)	0.0128 (18)	−0.0072 (14)
N1	0.0490 (13)	0.0441 (12)	0.0439 (13)	0.000	0.0054 (11)	0.000
O1	0.0563 (13)	0.0787 (15)	0.0725 (16)	0.000	−0.0153 (12)	0.000
O2	0.0762 (10)	0.0504 (8)	0.0721 (11)	−0.0099 (8)	−0.0117 (9)	0.0099 (7)

### Geometric parameters (Å, °)

**Table:**

C1—N1	1.463 (2)	C9—O2	1.369 (2)
C1—C6	1.507 (3)	C9—C10	1.376 (3)
C1—C2	1.554 (3)	C10—C11	1.385 (3)
C1—H1	0.9800	C10—H10	0.9300
C2—C3	1.502 (2)	C11—H11	0.9300
C2—C4	1.543 (3)	C12—O2	1.417 (2)
C2—H2	0.9800	C12—C13	1.510 (3)
C3—O1	1.208 (3)	C12—H12A	0.9700
C3—C2^i^	1.502 (2)	C12—H12B	0.9700
C4—C5	1.523 (3)	C13—C14	1.481 (3)
C4—H4A	0.9700	C13—H13A	0.9700
C4—H4B	0.9700	C13—H13B	0.9700
C5—C4^i^	1.523 (3)	C14—C15	1.510 (4)
C5—H5A	0.9700	C14—H14A	0.9700
C5—H5B	0.9700	C14—H14B	0.9700
C6—C11	1.382 (3)	C15—H15A	0.9600
C6—C7	1.390 (3)	C15—H15B	0.9600
C7—C8	1.374 (3)	C15—H15C	0.9600
C7—H7	0.9300	N1—C1^i^	1.463 (2)
C8—C9	1.384 (3)	N1—H1N	0.87 (3)
C8—H8	0.9300		
			
N1—C1—C6	112.25 (17)	O2—C9—C8	115.55 (18)
N1—C1—C2	109.31 (16)	C10—C9—C8	119.29 (19)
C6—C1—C2	112.33 (15)	C9—C10—C11	119.77 (18)
N1—C1—H1	107.6	C9—C10—H10	120.1
C6—C1—H1	107.6	C11—C10—H10	120.1
C2—C1—H1	107.6	C6—C11—C10	121.80 (19)
C3—C2—C4	106.96 (18)	C6—C11—H11	119.1
C3—C2—C1	107.76 (17)	C10—C11—H11	119.1
C4—C2—C1	115.76 (17)	O2—C12—C13	107.21 (19)
C3—C2—H2	108.7	O2—C12—H12A	110.3
C4—C2—H2	108.7	C13—C12—H12A	110.3
C1—C2—H2	108.7	O2—C12—H12B	110.3
O1—C3—C2	124.15 (12)	C13—C12—H12B	110.3
O1—C3—C2^i^	124.15 (12)	H12A—C12—H12B	108.5
C2—C3—C2^i^	111.7 (2)	C14—C13—C12	114.7 (2)
C5—C4—C2	113.74 (17)	C14—C13—H13A	108.6
C5—C4—H4A	108.8	C12—C13—H13A	108.6
C2—C4—H4A	108.8	C14—C13—H13B	108.6
C5—C4—H4B	108.8	C12—C13—H13B	108.6
C2—C4—H4B	108.8	H13A—C13—H13B	107.6
H4A—C4—H4B	107.7	C13—C14—C15	112.4 (2)
C4—C5—C4^i^	114.1 (2)	C13—C14—H14A	109.1
C4—C5—H5A	108.7	C15—C14—H14A	109.1
C4^i^—C5—H5A	108.7	C13—C14—H14B	109.1
C4—C5—H5B	108.7	C15—C14—H14B	109.1
C4^i^—C5—H5B	108.7	H14A—C14—H14B	107.9
H5A—C5—H5B	107.6	C14—C15—H15A	109.5
C11—C6—C7	117.37 (18)	C14—C15—H15B	109.5
C11—C6—C1	119.07 (18)	H15A—C15—H15B	109.5
C7—C6—C1	123.54 (17)	C14—C15—H15C	109.5
C8—C7—C6	121.36 (18)	H15A—C15—H15C	109.5
C8—C7—H7	119.3	H15B—C15—H15C	109.5
C6—C7—H7	119.3	C1—N1—C1^i^	111.3 (2)
C7—C8—C9	120.3 (2)	C1—N1—H1N	109.8 (9)
C7—C8—H8	119.8	C1^i^—N1—H1N	109.8 (9)
C9—C8—H8	119.8	C9—O2—C12	118.72 (17)
O2—C9—C10	125.15 (18)		
			
N1—C1—C2—C3	58.7 (2)	C1—C6—C7—C8	176.3 (2)
C6—C1—C2—C3	−176.04 (17)	C6—C7—C8—C9	0.5 (3)
N1—C1—C2—C4	−61.0 (2)	C7—C8—C9—O2	−179.7 (2)
C6—C1—C2—C4	64.3 (2)	C7—C8—C9—C10	1.5 (3)
C4—C2—C3—O1	−111.7 (3)	O2—C9—C10—C11	179.82 (19)
C1—C2—C3—O1	123.2 (3)	C8—C9—C10—C11	−1.6 (3)
C4—C2—C3—C2^i^	66.0 (3)	C7—C6—C11—C10	2.4 (3)
C1—C2—C3—C2^i^	−59.1 (3)	C1—C6—C11—C10	−176.39 (18)
C3—C2—C4—C5	−52.9 (2)	C9—C10—C11—C6	−0.4 (3)
C1—C2—C4—C5	67.2 (2)	O2—C12—C13—C14	179.6 (2)
C2—C4—C5—C4^i^	43.6 (3)	C12—C13—C14—C15	−177.8 (2)
N1—C1—C6—C11	−145.94 (19)	C6—C1—N1—C1^i^	172.73 (12)
C2—C1—C6—C11	90.4 (2)	C2—C1—N1—C1^i^	−61.9 (2)
N1—C1—C6—C7	35.4 (3)	C10—C9—O2—C12	−1.0 (3)
C2—C1—C6—C7	−88.3 (2)	C8—C9—O2—C12	−179.72 (19)
C11—C6—C7—C8	−2.4 (3)	C13—C12—O2—C9	−179.18 (19)

Symmetry codes: (i) *x*, −*y*+3/2, *z*.
